# Laparoscopic treatment of colonic endometriosis causing periodic abdominal pain and hematochezia: A case report

**DOI:** 10.1097/MD.0000000000036229

**Published:** 2023-11-24

**Authors:** Shiting Zhang, Xuelu Jiang, Peiyu Mao

**Affiliations:** a The First Affiliated Hospital of Zhejiang Chinese Medical University, Hangzhou, China; b Department of Gynecology and Obstetrics, The First Affiliated Hospital of Zhejiang Chinese Medical University, Hangzhou, China; c Department of Gynecology and Obstetrics, The First Affiliated Hospital of Zhejiang Chinese Medical University, Hangzhou, China.

**Keywords:** deep infiltrative endometriosis, indocyanine green, intestinal endometriosis, laparoscopic treatment, periodic abdominal pain

## Abstract

**Rationale:**

Endometriosis, a benign disease, has a malignant biological behavior and is highly prone to recurrence. Although gastrointestinal involvement is the most common site for extra-genital endometriosis, deep infiltrative endometriosis, which affects the mucosal layer, is very rare.

**Patient concerns:**

A 44-year-old woman with a 6-month history of recurring abdominal pain and Hematochezia. The patient visited several hospitals over the past six months and was suspected to have been diagnosed with a digestive disease, for which medication was ineffective, leading to a great deal of anxiety.

**Diagnoses:**

Colonic endometriosis.

**Interventions:**

After a thorough imaging evaluation and preoperative discussion, laparoscopic colonic endometriosis resection under indocyanine green indication was performed by gynecologists and gastroenterologists.

**Outcomes:**

After laparoscopic treatment, the patient's symptoms improved significantly, with occasional pain felt and no blood in the stool.

**Lessons:**

This case provides a rare example of sigmoid endometriosis causing periodic abdominal pain and Hematochezia. We report a clinical case to investigate the feasibility of an indocyanine green fluorescent contrast technique to guide the scope of surgery in laparoscopic deep infiltrative endometriosis surgery. In intestinal endometriosis surgery, indocyanine green fluoroscopy may indicate the lesion's precise localization.

## 1. Introduction

Endometriosis involving the intestine is a form of deep infiltrative endometriosis. Endometriotic lesions may infiltrate all layers of the intestinal canal, and it behaves similarly to a malignant tumor. The most common site of intestinal endometriosis is the rectosigmoid colon, with less common involvement of the sigmoid colon, cecum, appendix, and small intestine. Bowel endometriosis can lead to significant complications, including gastrointestinal bleeding, bowel obstruction, perforation, and malignant transformation. Surgery is the main treatment modality for deep infiltrative endometriosis (DIE), and early surgical intervention can improve symptoms and promote fertility.^[1]^ In intestinal endometriosis surgery, indocyanine green (ICG) fluoroscopy may indicate the precise localization of the lesion. It has been suggested that ICG fluoroscopy can help detect the presence of occult endometriotic lesions.^[2]^ ICG can bind to hemoglobin after intravenous injection and reveal the distribution of the vascular network, especially the neovascularization on endometriotic lesions in the near-infrared spectrum, thus showing the DIE lesions.^[2]^

## 2. Case presentation

We present a case of a 44-year-old woman with a 6-month history of recurring abdominal pain and Hematochezia. For the past 6 months, she had experienced 1 week of blood in her stool every month, the same as her previous menstrual cycle, with regular bowel movements the other 3 weeks. She described the bleeding as mild to moderate and noted that the blood typically mixed with stool and was dark red. These Hematochezia are accompanied by sharp lower abdominal pain, similar to past endometriosis. The woman underwent a total laparoscopic hysterectomy and bilateral salpingo-oophorectomy in 2020 for adenomyosis but preserved both ovaries. The only other surgical history was one lower uterine cesarean section. She had no family history of colorectal cancer or inflammatory bowel disease.

**Figure 1.**
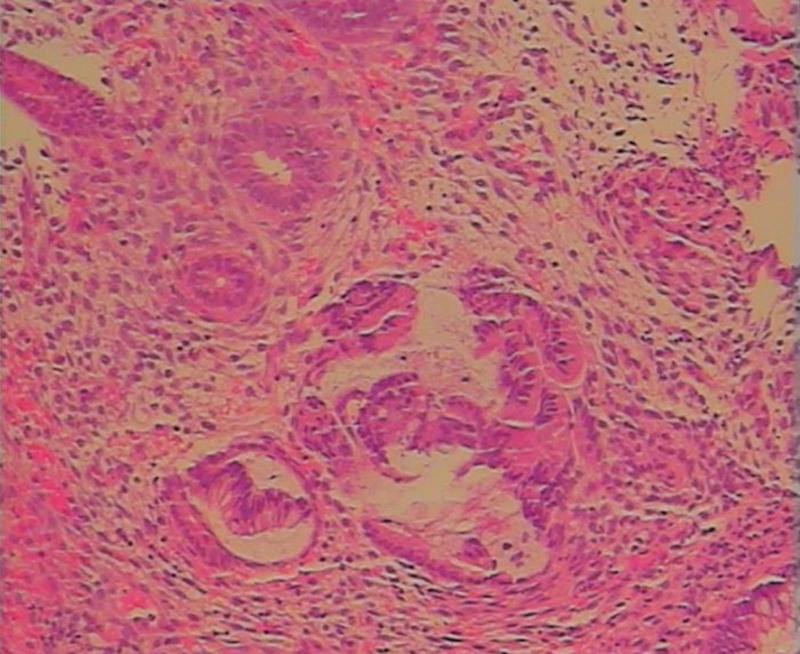
Picture of the pathology of colonoscopy at Sir Run Run Shaw Hospital, Zhejiang University.

The patient was diagnosed with ulcerative colitis and internal hemorrhoids in the gastroenterology department of several hospitals for cyclical abdominal pain and Hematochezia in the past 6 months – no medication improvement, leading to a chronic state of anxiety. The patient’s abdominal and pelvic enhancement computed tomography (CT) at a local hospital suggested a mid-sigmoid intestinal wall lesion suspected of cancerous. A colonoscopy at this hospital revealed colonic ulcers and rough mucosa on the surface of the sigmoid colon, and the pathology of the biopsy specimen suggested endometrium-like mesenchyme seen focally in the sigmoid colon, which combined with immunohistochemical findings, was consistent with endometriosis (Fig. 1). The patient was admitted to the hospital for further diagnosis and treatment.

**Figure 2.**
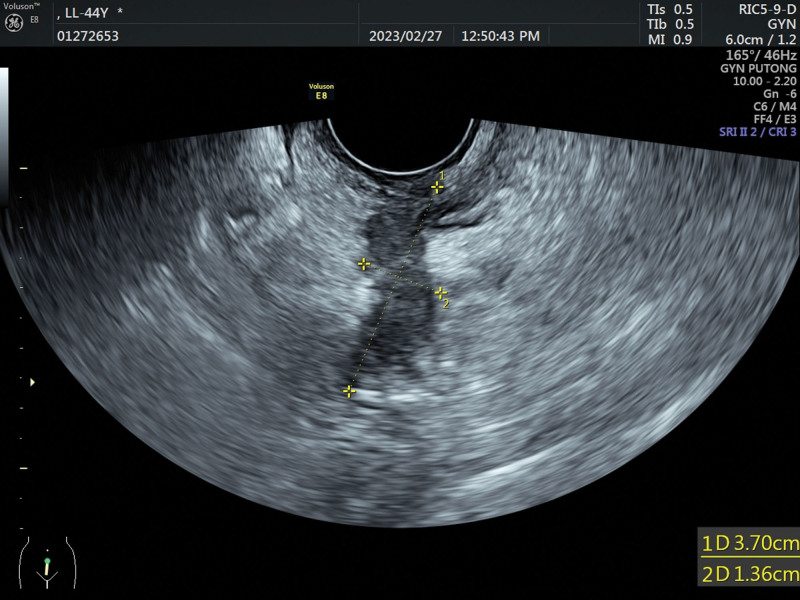
The ultrasound examination suggested a 3.7*1.4 cm hypoechoic cluster with a crab foot distribution to the canal wall.

**Figure 3.**
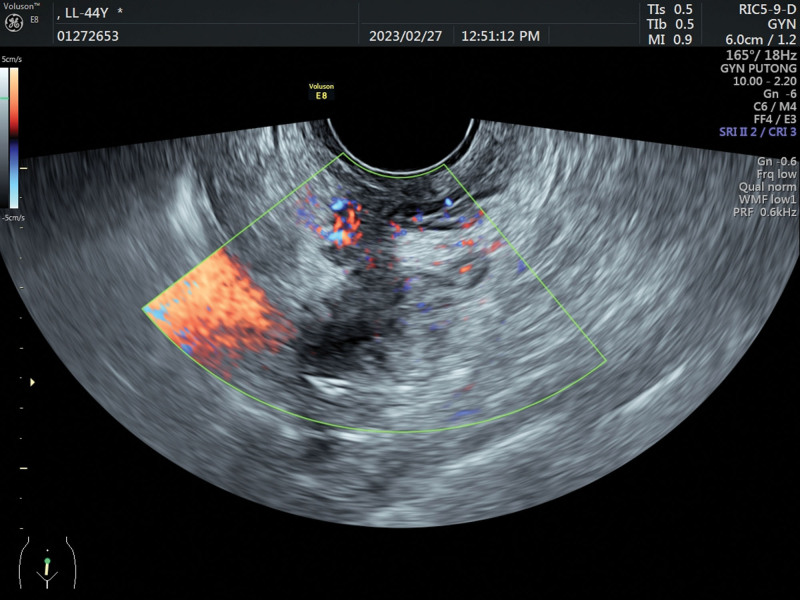
The lesion appears as a dendritic blood flow signal on color Doppler imaging.

**Figure 4.**
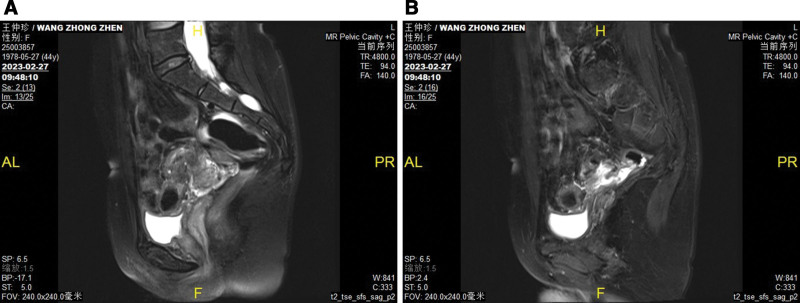
(A and B) MRI showed localized thickening of the intestinal wall in the sigmoid colon near the descending colon, and a lumpy abnormal signal shadow was seen next to the intestinal canal.

No significant abnormalities were found in blood routine, coagulation, liver, and kidney function. The tumor indicators were negative, and sex hormone levels were not at menopausal levels. The patient underwent transvaginal ultrasound, which revealed a 3.7*1.4 cm hypoechoic cluster with a crab foot distribution to the canal wall (Fig. 2) and a dendritic blood flow signal on color doppler flow imaging (Fig. 3). The lesion was considered as DIE (involvement of the muscular layer of the wall of the sigmoid colon). On radiological investigation, a contrast-enhanced CT scan of the chest and abdomen showed no primary or secondary lesions of the lungs, liver, or other visceral organs and no abdominal-pelvic lymphadenopathy. Since the CT scan was negative, the patient underwent pelvic magnetic resonance imaging to study better the lesion, which showed localized thickening of the intestinal wall in the sigmoid colon near the descending colon, and a lumpy abnormal signal shadow was seen next to the intestinal canal (Fig. 4A and B), and enhancement scans showed significant inhomogeneous enhancement. The magnetic resonance imaging report suggested that the lesion was closely related to the colonic intestinal canal, and a benign lesion was considered.

**Figure 5.**
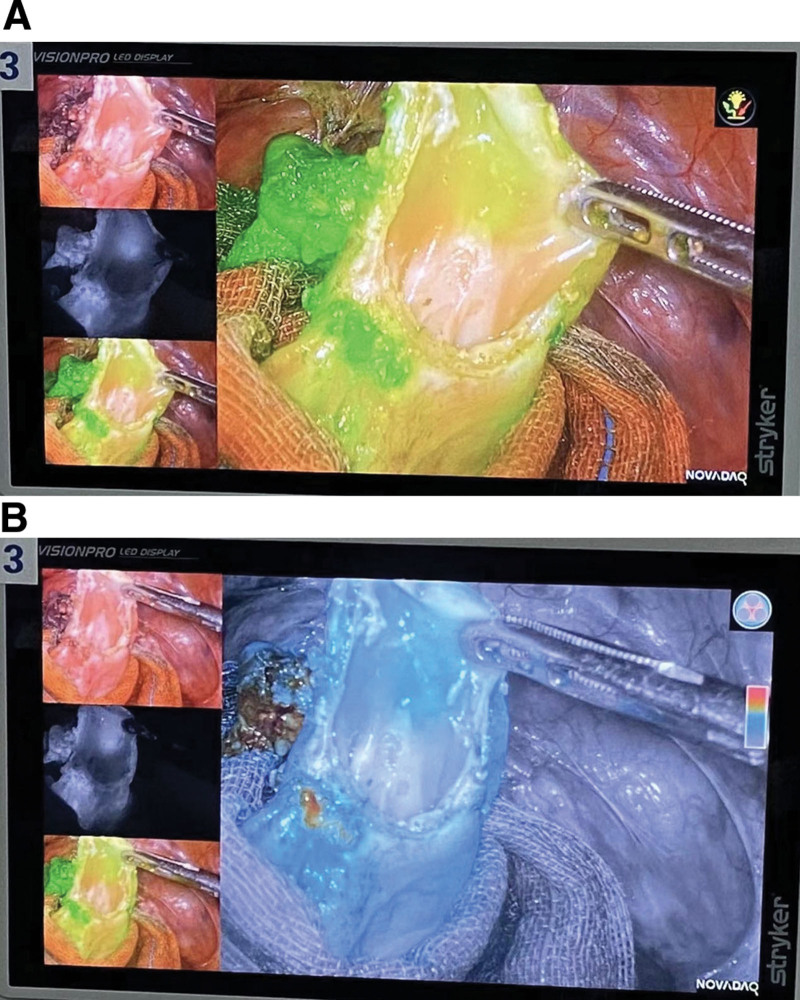
(A and B) Endometriotic foci of the sigmoid colon under indocyanine green visualization.

**Figure 6.**
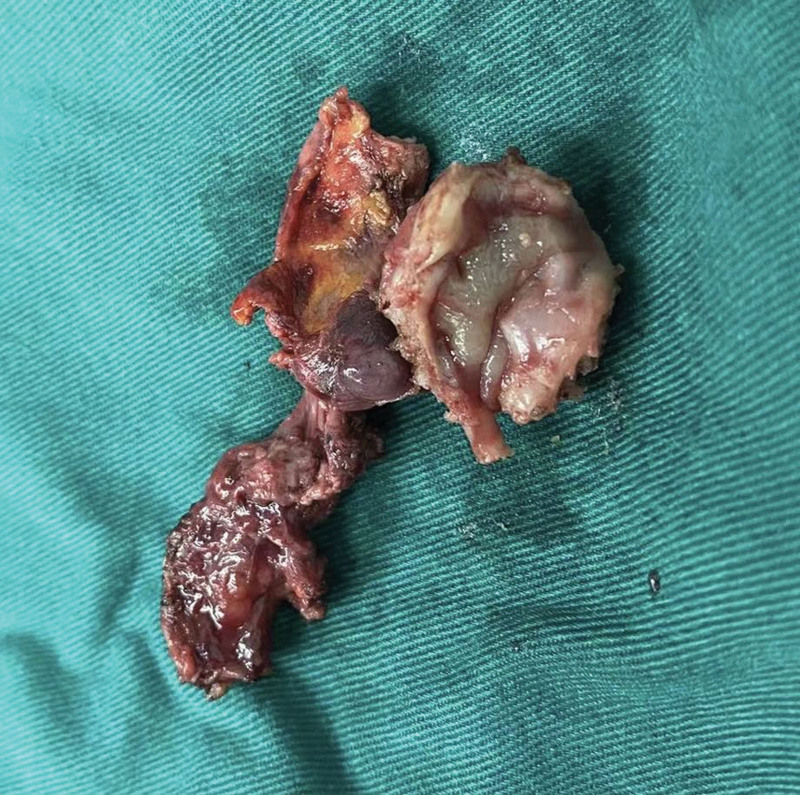
Surgically resected diseased sigmoid colon intestinal tube.

The patient felt that these symptoms were seriously affecting her quality of life and that conservative treatment with medication was not working for her, so she underwent laparoscopic surgery. After she provided written informed consent, a laparoscopy was performed. The agreement reached after full preoperative communication with the patient is that the specific surgical approach and the scope of surgery are decided according to the intraoperative exploration. If the intraoperative rapid cytopathology suggests malignancy, the area of surgery needs to be expanded. A team of gynecologists and gastrointestinal surgeons performed this complex surgery. Laparoscopically, the uterus was absent, and the sigmoid colon and part of the ileum were densely adherent to the left pelvic wall and pelvic floor, and the left ovary was wrapped in it. The ultrasonic knife carefully separates the adhesions between the intestinal canal and the pelvic wall. After separation, the left ovary was seen to be densely adherent to the sigmoid colon with partial fusion. ICG 15mg was injected intravenously and switched to fluorescence mode. Upon incision of the fusion, it was seen that the endometriotic lesion had invaded the mucosal layer of a segment of the sigmoid colon and appeared darker than the surrounding tissue under fluorescence visualization (Fig. 5A and B). Complete resection of the intestinal wall at the lesion (Fig. 6) and intestinal anastomosis were performed. The anastomosis was then tested, and the colonic inflation test was negative. Since the patient had no reproductive requirements, left ovarian endometriosis was taken as a left oophorectomy. No other apparent lesions were visible to the naked eye in the right ovary or elsewhere in the pelvis. Rapid intraoperative frozen section pathology suggested a lesion consistent with endometriosis.

The postoperative course was regular, with no complications or indices of inflammation. The bladder catheter was removed on the first postoperative day, and oral nutrition was restarted on the sixth postoperative day. The patient was discharged on postoperative day 10. The final surgical specimen was pathologically confirmed to be consistent with the intraoperative diagnosis of endometriosis, a benign condition. Immunohistochemical staining results: CK7(-), CD10(+), ER(+), PR(+), PAX-8(-), Ki-67 (proliferation band +). We recommend that patients receive 6 cycles of gonadotropin-releasing hormone agonists treatment with oral Chinese medicines that are effective for endometriosis. After laparoscopic treatment, the patient’s symptoms improved significantly, with occasional pain felt and no blood in the stool.^[3]^

## 3. Discussion

Endometriosis, a benign disease, has a malignant biological behavior and is highly prone to recurrence. The bowel is the extragenital site most frequently affected by endometriosis. 3.8% to 37.0% of patients with DIE have lesions involving the intestine.^[4]^ Generally speaking, hormonal medication’s effect on treating DIE is relatively weak. Early surgery is recommended for endometriosis that invades the intestine causing symptoms such as blood in the stool. If treatment intervention is delayed, the lesion will invade the entire intestinal canal and even turn into a frozen pelvis, so the opportunity for surgery is lost. For intestinal lesions, the surgical approach is divided into relatively conservative lesion debridement or lesion dissection and fairly radical resection of intestinal segments.^[5]^ It is generally considered that symptoms such as intestinal obstruction and blood in the stool are indications for bowel resection.^[6]^ Intraoperative application of ICG fluoroscopy helps to indicate endometriotic lesions and reduce damage to the normal intestine. It also allows the removal of visible lesions depending on the visualization site to reduce the recurrence rate after surgery.

The nonspecific clinical presentation of intestinal endometriosis is not easily distinguished from other diseases, so endoscopy is needed first to exclude lesions of the organ itself, especially malignant tumors.^[7]^ When intestinal endometriosis is highly suspected, a specialized treatment plan should be given by a multidisciplinary team based on the severity of the clinical symptoms, the assessment of imaging and the patient’s wishes.^[8]^

Deeper histopathological examinations and imaging examinations can be sought to improve diagnostic accuracy when intestinal symptoms change with the menstrual cycle. The pros and cons of different treatments must be thoroughly weighed to avoid the simple diagnosis of intestinal malignancy and inappropriate radical surgery that will result in permanent or temporary enterostomy and decreased life quality of patients.

## Author contributions

**Conceptualization:** Shiting Zhang, Peiyu Mao.

**Data curation:** Peiyu Mao.

**Formal analysis:** Xuelu Jiang, Peiyu Mao.

**Writing – original draft:** Shiting Zhang.

**Writing – review & editing:** Shiting Zhang, Xuelu Jiang, Peiyu Mao.
